# Supplementary figures and images for: Mechanism of LH release after peripheral administration of kisspeptin in cattle
Source: PLoS One. 2022 Dec 2;17(12):e0278564. doi: 10.1371/journal.pone.0278564 (PMC9718405; doi:10.1371/journal.pone.0278564)

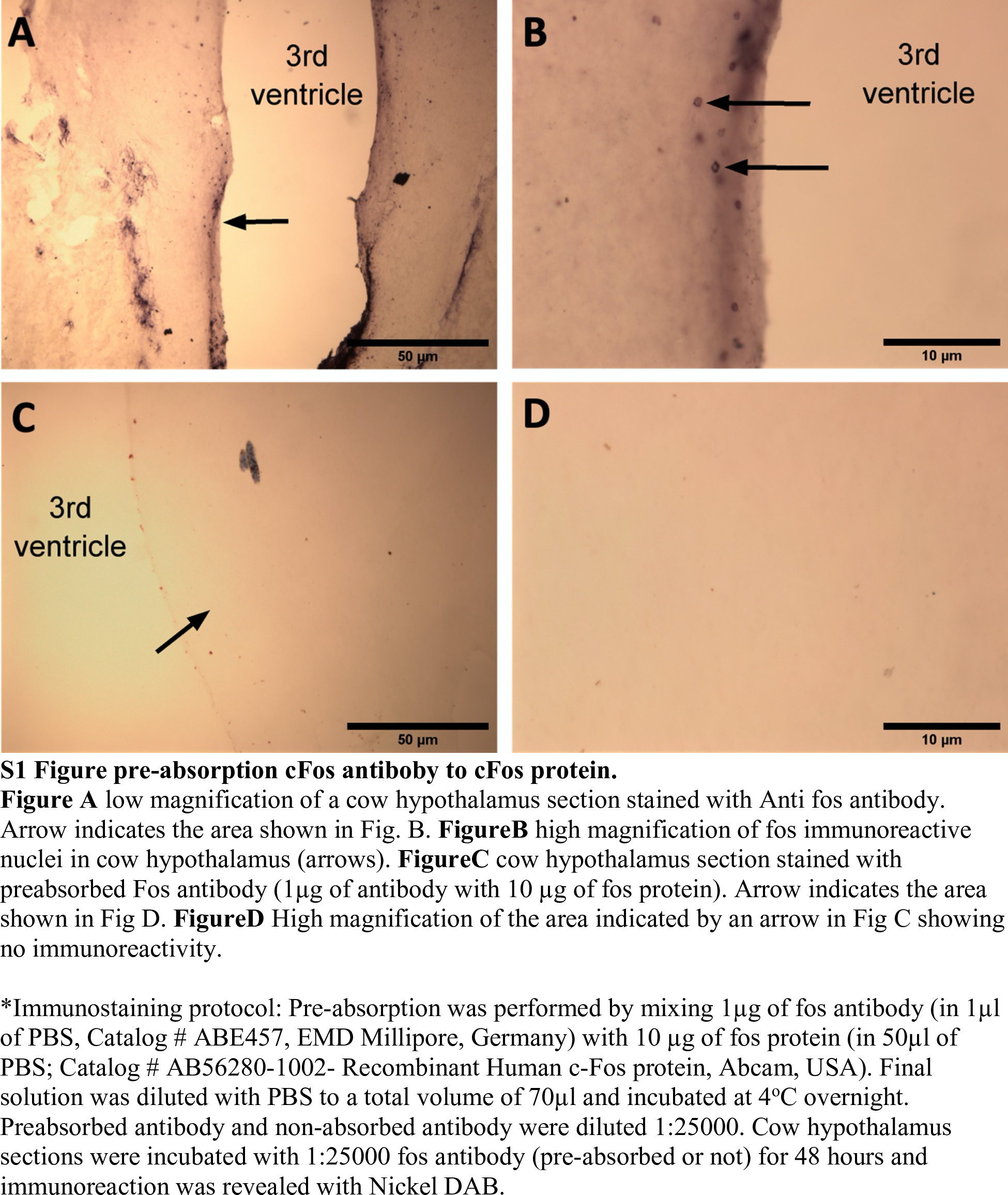

Supplement: S1 Fig — Fig A low magnification of a cow hypothalamus section stained with Anti fos antibody. Arrow indicates the area shown in Fig B. Fig B high magnification of fos immunoreactive nuclei in cow hypothalamus (arrows). Fig C cow hypothalamus section stained with preabsorbed Fos antibody (1μg of antibody with 10 μg of fos protein). Arrow indicates the area shown in Fig D. Fig D High magnification of the area indicated by an arrow in Fig C showing no immunoreactivity. (TIF) [file pone.0278564.s001.tif]
