# Supplementary material for: Mechanism of LH release after peripheral administration of kisspeptin in cattle
Source: PLoS One. 2022 Dec 2;17(12):e0278564. doi: 10.1371/journal.pone.0278564 (PMC9718405; doi:10.1371/journal.pone.0278564)
Supplement: Attachment — Submitted filename: Response to reviewers.docx [file pone.0278564.s002.docx]

Reviewer #1:

1. Line 44 and throughout the manuscript: Since the ligand for the GPR54 has been identified, GPR54 has been renamed as Kiss1R. I suggest the authors could briefly mention here that GPR54 and Kiss1R are the same receptor and thereafter refer to the receptor as Kiss1R and not GPR54.

Thank you for the suggestion. Proposed change was done.

2. Line 48: “Furthermore, GnRH and kisspeptin immunoreactive cells are located in close association in the preoptic area and arcuate nucleus of the hypothalamus in mice, primates, sheep [7-9] and cattle [10].”. This statement is not technically correct. Are the authors referring to cell bodies or neuronal projections? In rodents, GnRH neurons are limited to the POA. In ruminants and primates, GnRH neurons migrate caudally to the anterior portions of the hypothalamus. In rare occasions, GnRH neurons can be found in the ARC in cattle, but the large majority of GnRH neurons is located in the POA and in the anterior hypothalamic area (AHA). If authors are referring to GnRH neuronal projections, those are also not commonly present in the ARC. In all species mentioned above, the large majority of GnRH projections are in the zona externa of the median eminence. Obviously, a large amount of kisspeptin projections is also observed in the median eminence, particularly in the internal zone.

Thank you for the detailed explanation. The sentence was change to “ Furthermore, GnRH and kisspeptin immunoreactive cells are located in the preoptic area and in the hypothalamus in mice, primates, sheep [7-9] and cattle [10], and its projection are detected in close association in the median eminence”.

3. Line 81: “…to stimulate GnRH neuronal cell bodies, acts on GnRH nerve terminals…” Remove “or”

It was removed

4. Line 125: Please explain how the Kp10 dose (15 mg) was determined? Extrapolation from other species? Previous studies in cattle?

The dose was determined based on our previous studies in cattle (Leonardi et al., 2018, Leonardi et al., 2020); “The dose and frequency of treatment was determined based on our previous studies in cattle [16, 17]”.

5. Line 219: While properly explained in the Methods, the design for Expt. 2 is somewhat hard to follow. A diagram figure with the experimental timeline would help readers to better understand the experimental design for study 2.

Thank you for the suggestion. Timeline diagram is now included as figure 1 in the revised manuscript

6. Line 223: PGF2alpha: Dose? Manufacturer? Mode of administration?

The sentence was add “(500 μg cloprostenol im, Estrumate, Merck Animal Health, Kirkland, QC, Canada)”

7. Line 322: Remove underline between “sections” and “from”

It was removed

8. Line 323: what do authors mean with “association between kisspeptin and GnRH nerve terminals”? I suggest the authors to replace “association” with “close-appositions” or another technical term.

It was replaced

9. Line 392: remove “but” after “cows”

It was removed

10. Line 412: “Currently, this approach is not technically feasible in cattle but a similar mechanism of action of kisspeptin is plausible.” That is not necessarily correct since third ventricle cannulation can be used in cattle to collect CSF and characterize secretion of GnRH and other hypothalamic neuropeptides. Please see papers by G Williams et al. (Biology of Reproduction, 1998; 59(3), 676-683 among other papers by the same group).

We were referring to hypophysial portal blood circulation. It is clarified in the revised manuscript.

11. Line 434: replace “interesting” with “interestingly”

It was fixed

12. Line 442: “Kisspeptin neurons are clustered in two locations in cows – a cranial population in the medial preoptic area and second aggregation in the caudal portion of arcuate nucleus [50]” This is not technically correct. In cattle, majority of kisspeptin neurons are located in the rostral and middle regions of the ARC (see Tanco et al., 2016 PeerJ and Alves et al., BOR 2015).

It was fixed

The sentence was added “and in cows the majority of kisspeptin neurons are detected in the rostral and middle regions of the arcuate nucleus [10, 51].”

Reviewer #2:

1. The sample size in Experiment 1 is very low. Together with the variability of cFos between animals and greater than 50% more GnRH neurons identified in the treatment group, could the authors accurately conclude there is no increased activity of GnRH neurons with kisspeptin administration?

We understand the reviewer inquiry, however the number of GnRH cells that co-expressed cFOS is very low in both treatments. For this reason we believe that there is no effect of kisspeptin on the cFos activation.

2. Do the authors think the use of lactating cows with low LH response (2 of 3) may be a confounding factor in the cFos experiment?

Also there was not difference in the LH concentration among animals in the pre-treatment period. So, in our opinion, this is no confounding due to lactation in this experiment. We believe that this may be attributed to individual variation post-treatment which could happen in frequent-bleeding studies.

3. Please include references to validated use of kisspeptin and cFos antibodies in bovine hypothalamic tissue or provide details of preabsorption experiments herein. Lack of primary is not sufficient to determine antibody specificity. Also include details on these important controls in either the methods section or results section.

We performed the absorption validation as suggested. This is now included as supplementary data with analyses and images of pre-absorption cFos antiboby to cFos protein.

4. italicize the use of in vivo, in vitro, ex vivo, etc throughout text of manuscript

It was changed

5. Line 383- replace "neuron" with "cell bodies" given that you are arguing the neuron is activated at the terminal to release GnRH but not at the cell body

It was replaced

6. Make sure to include Cetrorelix + Kp10 in Table 3 and Figure 4

Treatment of this group is described in the table and figure titles. So, we opted to keep the group name as cetrorelix. We hope that is OK with the reviewer.
